# Therapy-type related long-term outcomes in mucopolysaccaridosis type II (Hunter syndrome) – Case series

**DOI:** 10.1016/j.ymgmr.2021.100779

**Published:** 2021-06-26

**Authors:** Mojca Zerjav Tansek, Jana Kodric, Simona Klemencic, Jaap Jan Boelens, Peter M. van Hasselt, Ana Drole Torkar, Maja Doric, Alenka Koren, Simona Avcin, Tadej Battelino, Urh Groselj

**Affiliations:** a Department of Endocrinology, Diabetes, and Metabolic Diseases, University Children's Hospital, UMC Ljubljana, Ljubljana, Slovenia; b Faculty of Medicine, University of Ljubljana, Ljubljana, Slovenia; c Department of Child Psychiatry, University Children's Hospital, UMC Ljubljana, Ljubljana, Slovenia; d Department of Pediatric Immunology, Wilhelmina Children's Hospital, University Medical Center Utrecht, Utrecht, the Netherlands; e Stem Cell Transplantation and Cellular Therapies Program, Department Pediatrics, Memorial Sloan Kettering Cancer Center, New York, NY, USA; f Department of Metabolic Diseases, Wilhelmina Children's Hospital, University Medical Center Utrecht, Utrecht University, Utrecht, the Netherlands; g Department of Pediatric Hematology and Oncology, University Children's Hospital, UMC Ljubljana, Ljubljana, Slovenia

**Keywords:** MPS, mucopolysaccharidosis, ERT, enzyme replacement therapy, HSCT, hematopoietic stem cell transplantation, CNS, central nervous system, LSD, lysosomal storage disease, I2S, iduronate-2-sulfatase, GAG, glycosaminoglycans, ENT, ear nose and throat specialist, MPS, Mucopolysaccharidosis type II, Hunter syndrome, Hematopoietic stem cell transplantation, HSCT, enzyme replacement therapy

## Abstract

Mucopolysaccharidosis type II (MPS II, Hunter syndrome) is a rare, X-linked recessive multisystem lysosomal storage disease due to iduronate-2-sulfatase enzyme deficiency. We presented three unrelated Slovenian patients with the severe form of MPS II that received three different management approaches: natural course of the disease without received specific treatment, enzyme replacement therapy (ERT), and hematopoietic stem cell transplantation (HSCT). The decision on the management depended on disease severity, degree of cognitive impairment, and parent's informed decision. The current benefits of MPS II treatments are limited. The lifelong costly intravenous ERT brings significant benefits but the patients with severe phenotypes and neurological involvement progress to cognitive decline and disability regardless of ERT, as demonstrated in published reviews and our case series. The patient after HSCT was the only one of the three cases reported to show a slowly progressing cognitive development. The type of information from the case series is insufficient for generalized conclusions, but with advanced myeloablative conditioning, HSCT may be a preferred treatment option in early diagnosed MPS II patients with the severe form of the disease and low disease burden at the time of presentation.

## 1 Introduction

Mucopolysaccharidosis type II (MPS II) (Hunter syndrome, OMIM 309900) is an ultra-rare, X-linked multisystem lysosomal storage disease (LSD) due to deficiency of the iduronate-2-sulfatase (I2S) enzyme, caused by spectrum of variants in the *IDS* gene [1]. The cumulative incidence of MPS II is estimated at 0.64 cases per 100,000 births [2,3].

I2S deficiency results in the accumulation of glycosaminoglycans (GAG) in lysosomes throughout nearly all cell types and tissues, including the oropharynx, upper respiratory tract, heart, liver and spleen, bones, and joints, meninges, and central nervous system [1].

Characteristic signs and symptoms of the early severe MPS II phenotype usually develop between 18 months and four years of age and include inguinal and umbilical hernia, frequent respiratory infections that lead to hearing impairment, macrocephaly, macroglossia, coarse facial features, bone dysplasia with joint stiffness, and claw-like shaped hands. During the first years of life, patients can exhibit overgrowth in weight and height but deteriorate to short stature as the disease progresses [4]. Sleep apnea can occur. Behavioral problems and loss of acquired developmental milestones become evident [[3], [4], [5]]. Deterioration to severe cognitive impairment, progressive neurodegeneration, and death occurs at the end of the first or the second decade of life [4]. Cardiac involvement is frequently present, and cardiac valve disease is reported in over half of MPS II patients [6].

The genotype-phenotype correlation is unpredictable, especially in the early phase of the disease; however, deletions and gene rearrangements in *IDS*, which account for up to 25% of MPS II cases, always result in a severe phenotype [7]. The unpredictable correlation additionally jeopardize the conclusions from the case reports of MPS II patients.

Quantitative and qualitative analysis of urinary GAGs is helpful as a preliminary MPS screening test; however, demonstration of I2S deficiency and normal level of at least one sulphatase to exclude SUMF1 deficiency (sulfatase modifying factor 1) in dry blood spot, leukocytes, fibroblasts, or plasma is necessary for MPS II diagnosis confirmation in a male proband. Molecular genetic testing of the *IDS* gene is sometimes required for diagnosis confirmation [3].

Exogenous enzyme replacement therapy (ERT) uses the technique of cross correction by delivering the recombinant enzyme. The disease-specific ERT with enzyme idursulphase is available since 2007 in the European Union [8]. Total urine GAGs concentrations were used as markers of the ERT efficacy in most studies, but GAGs levels alone do not accurately reflect the biochemical response to ERT [8]. The production of antibodies against the recombinant enzyme, a suboptimal dosage of the enzyme, a poor enzyme penetration to connective tissues, and other currently not yet understood factors influence the therapeutic effects in single individuals [9].

One of the most common metabolic indications for allogeneic hematopoietic stem cell transplantation (HSCT) is MPS type I (Hurler syndrome). However, the efficacy of HSCT in MPS II is still debated [10]. Evidence from the systematic analysis of MPS II treatment outcomes is changing the presumptions [11]. HSCT seems to be more effective than ERT for MPS II in a wide range of disease manifestations and is considered as a treatment option for this condition [11]. Engrafted donor leucocytes secrete the enzyme, which is taken up by deficient host cells in a process known as a cross correction, forming the basis for the correction of metabolic disorder by transplantation.

We aimed to present clinical and genetic characteristics of the only three unrelated Slovenian patients with MPS II diagnosed so far, all of them having the severe form of the disease. Early manifestations and the course of the condition concerning the used treatment approach are presented.

## 2 Case presentations

### 2.1 Case 1

26-months old boy was referred to a metabolic specialist due to suspected lysosomal storage disease. Family history was unremarkable. He was born after an uneventful pregnancy at term with a birth weight of 3550 g and a length of 53 cm. The development was timely in the first year; he was sitting at 7.5 months and walked unsupported at 15 months of age. Until the age of 22 months, he had had right inguinal hernioplasty, operative repair of right hydrocele, tympanoplasty, and adenoidectomy. A heart murmur was recorded for the first time during the treatment of dehydration and rotavirus infection at 21 months of age. Ultrasound of the heart showed a mild mitral regurgitation, together with left ventricular hypertrophy and compromised systolic function with an ejection fraction of 58%. At follow-up, myxomatous degeneration of the mitral valve with partial prolapse and progressive regurgitation was described. At 26 months, he was seen by a neurologist because of a developmental delay and stumbling gait. Macrocephaly, macroglossia, coarse facial features, swelling-like shaped fingers were described (Table 2).

Polysomnography showed a mild obstruction hypopnea in sleep. The auditory evoked brainstem potentials test showed a moderate conductive hearing loss of the right ear and mild sensorineural impairment of the left ear. Psychological tests showed impaired cognitive abilities and autistic-like behavioral features with stereotypic behavior and absent eye contact, and impaired social interactions.

Quantified urine GAGs were elevated, and the electrophoresis of GAGs indicated MPS I, II, or multiple sulphatase deficiency (in all the three cases reported, GAGs were determined by method previously described by Dembure et al. [12]). Undetectable levels of iduronidase activity and low activity of arilsulphase B and arilsulphase A suggested multiple sulphase deficiency. The repeated test of enzyme activities confirmed isolated I2S deficiency and normal levels of other lysosomal enzymes together with normal genotyping of *SUMF1* gene (sulfatase modifying factor 1) (in all the three cases reported, the IDS activity in peripheral blood leukocytes was determined by method previously described by Voznyi et al. [13]). He was hemizygous for a known mutation p. Ser333Leu in exon 7 of the *IDS* gene, which is indicative of a severe phenotype of MPS II [8]. Molecular-genetic analyses of *IDS* gene in all three cases reported were performed by method previously described by Dvorakova et al. [14]. The boy was not a candidate for HSCT because of the progressed disease. ERT with idursulphase was recommended, but parents refused all further follow-up or therapies. There was a significant decline in the boy's heart function with thickening of the mitral valve and severe regurgitation in the third year of life. Surgical treatment of the heart condition was not indicated; he only received symptomatic treatment with angiotensin-converting-enzyme inhibitor He died at the age of 3.5 years because of cardiorespiratory failure during an episode of pneumonia.

### 2.2 Case 2

35-months old boy was referred for metabolic evaluation by the pediatric neurologist because of clinical suspicion of lysosomal storage disease. Family history revealed that the boy's uncle on the maternal side of the family had an unusually shaped head and died of an unknown cause as a teenager. The boy was born at term, after an uneventful pregnancy and perinatal history with appropriate birth measurements. In the first year, general muscular hypotonia was noticed, but the early developmental milestones were reached at a proper age; he was sitting unsupported at seven months, used the first words at one year of age, and walked independently at 15 months of age. After joining kindergarten at 18 months of age, he had recurrent middle ear infections and upper respiratory tract infections with chronic rhinitis, which led to adenoidectomy at 27 months. This was associated with a temporary improvement of his hearing and rhinitis, but in four months, his parents noticed additional speech regression and hearing impairment. The auditory brainstem response test was normal. Another adenoidectomy was performed. By 34-months, he was referred to a pediatric neurologist because of speech delay, less constructive play patterns with a lack of understanding of social concepts, and poor concentration. The neurologist noticed facial dysmorphism and developmental regression, which indicated a possible diagnosis of MPS.

Quantified urine GAGs were elevated (57 mg/mmol creatinine, reference value <20 mg/mmol) [12]. The diagnosis of MPS II was confirmed by I2S enzyme deficiency and disease-causing *IDS* gene variation (c.251 G > C; p.C84S), a known variant without measurable enzymatic activity [[13], [14], [15]] (Table 1).

**Table 1:** The diagnostic features of patients.

	Case 1	Case 2	Case 3
Age at the first symptoms	21 months	18 months	8 months
Indications for the referral	Developmental delay, speech regression, heart valve disease, stumbling walk	Speech regression, developmental regression, and delay; hearing impairment	Developmental delay, sleep apnoea, repeated respiratory infections
Age at diagnosis	29 months	35 months	16 months
Genotype	p.S333L adjacent to the active site and changes the core structure	p.C84S active site mutations with no enzyme activity	Hemizygotic deletion and inversion of 5′ gene

At the first presentation to the metabolic specialist, the boy had coarse facial features, macrocephaly, macroglossia, short neck, hepatosplenomegaly, and umbilical hernia (Table 2). Heart ultrasound revealed left ventricular hypertrophy with mitral regurgitation. Polysomnography recorded obstructive sleep hypopnea syndrome. EMG test confirmed a developing carpal tunnel syndrome. The ophthalmologist reported central opacities of the cornea.

**Table 2:** Clinical characteristics of patients at onset and at last follow-up.

	Case 1	Case 2	Case 3
Signs and symptoms at the onset / before the treatment			
Clinical features	Coarse facial features, small umbilical hernia, hepatosplenomegaly	Coarse facial features, macrocephaly, umbilical hernia hepatosplenomegaly	Macrocephaly, macroglossia umbilical hernia hepatosplenomegaly
Height	96.5 cm (96.P.)	111 cm (51.P.)	83 cm (94.P.)
Weight	15.9 kg (96.P.)	34 kg (97.P.)	12 kg (92.P.)
Head circumference	52.2 cm (97.P.)	Not available	49 cm (93.P.)
Polysomnography and ENT	Normal polysomnography, hearing impairment, recurrent respiratory/ear infections	Obstructive hypopnea, hearing impairment, and recurrent ear/nose infections	Obstructive apnea/hypopnea, hearing loss needing a hearing aid, recurrent respiratory/ear infections
Previous operations	Bilateral inguinal hernia repair, hydrocele repair, adenoidectomy, grommet insertion	Adenoidectomy, grommet insertion	/
Cardiovascular symptoms	Dilatative cardiomyopathy, mitral valve myxomatous degeneration, and prolapse, moderate to severe mitral insufficiency	Mild aortic valve insufficiency, myxomatous degeneration of mitral valve with mild mitral insufficiency	Mitral valve prolapse, mild mitral insufficiency
Skeletal features	Dysostosis multiplex Flexion contractures of knees, elbows, and shoulders	Normal body height, mild dysostosis multiplex	Normal body height, mild dysostosis multiplex, slight thoracolumbar kyphosis
Neurocognitive functioning	Moderate intellectual disability, autistic spectrum disorder	Severe intellectual disability mild carpal tunnel syndrome	Mild developmental delay


Signs and symptoms at the last follow-up / after the treatment			
	Died at 3.5 years with cardiorespiratory failure; no specific therapy was applied	After 10 years of enzyme replacement therapy (at 14 years of age)	Four years post-HSCT (at 6 years of age)
Features and signs	/	Coarse facial features, no hepatosplenomegaly	Normal facial features, no hepatosplenomegaly
Height	98 cm (75.P.)	144 cm (<3.P.)	130.2 cm (98.P.)
Weight	15 kg (89.P.)	47 kg (15.P.)	35 kg (>99.P.)
Head circumference	Not available	Not available	Not available
Polysomnography and ENT	/	Normal polysomnography, no recurrent respiratory/ear infections	Normal polysomnography, no recurrent respiratory/ear infections, hearing loss with hearing aids
Cardiovascular symptoms	/	Aortic regurgitation with left ventricular dilatation and reduced ejection fraction, mild mitral regurgitation	Mild mitral insufficiency without progression
Skeletal features and mobility	/	short stature, progressive features of dysostosis multiplex with contractures, unsteady gait	Normal body height, no progression in thoracolumbar kyphosis, mild dysostosis multiplex mild motor developmental delay (immature fine motor skills)
Sphincter control	/	Not achieved	Achieved
Neurocognitive functioning	/	Profound intellectual disability without meaningful communication	Moderate intellectual disability, progress in cognitive development, vocabulary with 50 words, two-word sentences, short attention span

**Fig. 1:**
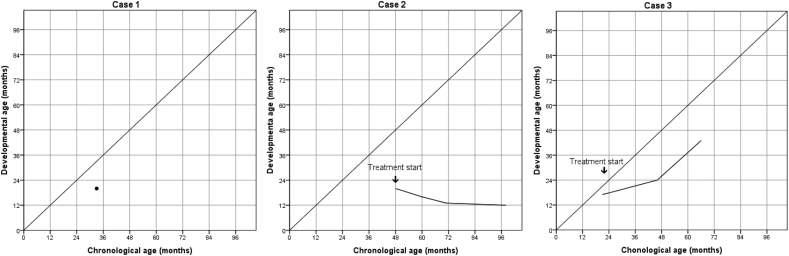
Growth curve of the developmental age of each patient. Developmental age was assessed by Bayley Scales of Infant and Toddler Development-III (Case 1 and 2) or Bayley Scales of Infant and Toddler Development-II and Snijders-Oomen nonverbal intelligence test (Case 3).

ERT with idursulphase (Elaprase®) started at four years of age in the recommended dose 0.5 mg per kg of body weight every week as an intravenous infusion administered over a period of three hours. ERT is ongoing during the last ten years; he experienced a few mild infusion-related reactions during the first year of ERT with skin rash and pruritus. The neutralizing antibodies for idursulphase were not detectable. Parents reported a drastic drop in the frequency of respiratory infections, no deterioration in mobility, and improved quality of life during the first three years of ERT. The heart hypertrophy started to progress during the fourth year of ERT. Hepatomegaly normalized at ten years of age. A progressive decline in cognitive functions has been observed (Fig. 1). There is no meaningful verbal communication. Sphincter control was never achieved. The flexion contractures of large joints and fingers are present, he is still walking independently at the age of 14 years, but the gait is unsteady. Hemodynamically significant aortic regurgitation with left ventricular dilatation and reduced ejection fraction was reported at the last heat examination at 14 years (Table 2).

### 2.3 Case 3

14-months old boy was referred to a metabolic specialist due to clinical signs and symptoms suspicious of lysosomal storage disease. Developmental delay and facial dysmorphism were the presenting signs (Table 2). Family history was unremarkable. He was born at term after a normal pregnancy with a birth weight of 3710 g and length of 52 cm. The neonatal period was uneventful. He achieved developmental milestones timely for the first eight months; afterward, he stopped gaining new skills. Frequent upper respiratory tract and middle ear infections were reported. Parents noticed obstructive sleep apnea at 11 months of age.

At presentation, coarse facial features, macroglossia, inspiratory stridor, umbilical hernia, hepatosplenomegaly, and hypotonia were noticed (Table 1). Social contact and walking were appropriate for the age. ENT specialist described enlarged adenoids, and obstructive sleep hypopnea syndrome was confirmed by polysomnography. The skeletal X-rays indicated incipient dysostosis multiplex. Mitral valve prolapse with minor mitral regurgitation was evident on heart ultrasound examination. The ophthalmological evaluation found no abnormalities (Table 2).

Quantified GAGs in urine were elevated, and electrophoresis indicated MPS type I or II. The diagnosis of MPS II was confirmed by an undetectable isolated I2S enzyme deficiency. A hemizygotic deletion and inversion of the 5′ part of the *IDS* gene confirmed MPS II (Table 1).

Regarding the age and spared cognitive functions, the boy was presented as a suitable candidate for HSCT, which was performed at 22 months in UMC Utrecht, the Netherlands. While awaiting HSCT, he received eight applications of ERT 0.5 mg idursulfase per kg of body weight every week without adverse or infusion related reactions. The neutralizing antibodies for idursulphase were not assessed. The HSCT procedure with unrelated cord blood (match grade: 6/6; 10/10 high resolution) transplantation and Thymoglobuline (10 mg/kg, in 4 days), Busulfan (myeloablative exposure of 90 mg*h/L) with Fludarabine 160 mg/m2 (over 4 days); pharmacokinetics-targeted myeloablative conditioning together with post-transplantation period went without complications. His regular yearly check-ups at UMC Utrecht and UMC Ljubljana have shown unrefined and immature, nonetheless qualitatively normal motor skills. There is still a developmental cognitive and speech delay; he is hyperactive and has a short attention span (Table 2, Fig. 1).

## 3 Discussion

Combining data from case reports in systematic reviews is essential to obtain better insight into the course of the ultra-rare diseases, including MPS II [16,17]. Three patients with a severe form of MPS II were presented. They were between 8 and 21 months of age at the first presentation. Still, they differed in the disease burden that led to different management approaches: no specific treatment with the natural course of the disease observed, a long-lasting ERT and HSCT. The difference in clinical history could not be explained by the genetic variants underlying the disorder (Table 1): disease-causing gene variants in all three cases were already published, and they predicted severe phenotype without measurable enzyme activity [7,15]. Genotype-phenotype correlation was identified in several missense mutations and gene rearrangements but the phenotype and clinical outcomes are sometimes poorly predicted in MPS II patients, indicating the possible role of genetic background [14]. The conclusions from the case reports are drawn with additional speculations. The presenting symptom in the reported cases was a consistently delayed development (Fig. 1) with variable additional specific abnormalities: a cardiac valve disease, stumbling walk (Table 2).

The decision on the management depended on disease severity and its progression velocity, degree of cognitive impairment, the availability of ERT, and parent's agreement with the therapy. The caregiver's role in the treatment decision-making process represents a complex ethical dilemma [18]. The burden of the disease for the parents is extensive in MPSs: 69% of working caregivers reported a negative impact on their ability to conduct work tasks, and 54% of caregivers were not employed to be able to take care of the child full-time [19].

The patient described as Case 1 was referred late, with progressive neurologic deterioration and cardiac abnormalities at baseline. The progressed phase of the disease was not indicating HSCT as a treatment option, and the parents understood the limitations of the ERT. The family made an informed decision to decline the available therapy due to the poor prognosis and burden of the ERT applications for the poorly cooperative boy.

The cardiac valve disease is present in over half of the patients with MPS II [4,6,20], and the valve pathology is progressive regardless of received ERT [21]. The deterioration of the valve regurgitation and left ventricular dysfunction are expected in the natural course of the disease; however, they are not described as a cause of death in patients before five years of age [4,22]. We speculated that disease-related airway obstruction and progressed heart disease contributed to irreversible heart failure during pneumonia with sepsis complications. The parents accepted the complication as a part of the life-threatening condition; the boy arrived at the hospital at a late phase of sepsis deterioration.

The lifelong continuous weekly intravenous ERT brings significant benefits, including improved walking ability, reduced hepatosplenomegaly, ameliorated respiratory symptoms, overall life-quality improvement, and improved survival [8,23,24]. The cost-effectiveness, accessibility, and limited biodistribution into bone, cartilage, heart, and especially non-permeability to CNS should be discussed when deciding for ERT [9,23,25]. Patients with genotype linked to severe phenotypes and symptomatic patients with severe neurological involvement are expected to progress to cognitive decline and disability regardless of regular ERT [24]. Additionally, ERT appears to have minimal impact on hearing loss, carpal tunnel syndrome, and progression of cardiac valvular disease [24].

The boy presented as Case 2 was four years old and the oldest of our cohort at presentation, with evident cognitive impairment. At that age, the plateau or gradual cognitive decline is expected, according to natural history studies [26,27]. With the genotype-phenotype relationship, a severe course of the disease was expected, and limited effectiveness of ERT was predicted. His cognitive abilities continued to decline after ERT (Fig. 1), reaching profound cognitive disability without meaningful communication and eye contact at the age of ten (Table 2, Fig. 1). The patient's condition agrees with the evidence from other long-term follow-up studies in severe MPS II receiving ERT [3,24,28]. The improved overall quality of life of patients and their families is a crucial goal of the treatment and must be considered when deciding to continue ERT [29,30]. Lampe et al. speculate introduction of ERT in the first year of life predicts a less severe clinical course and improved clinical outcome [31]. However, the summarized evidence seems insufficient to support this premise [20]. Nonetheless, ERT is the recommended treatment option for MPS II since it is less invasive and has fewer risks than HSCT [32].

HSCT decreases urinary and blood GAGs more than ERT and normalizes or stabilizes the I2S enzyme activity in leukocytes [11]. HSCT improves somatic and skeletal symptoms of the disease and slows the overall disease progression but might not improve CNS manifestations of the disease if brain involvement is progressed at baseline [11,33,34]. HSCT is the preferred treatment option for MPS II in Japan, China, and Brazil [30,32]. The predictors of outcome after HSCT are the timing of the treatment, the scheme of myeloablative conditioning, and reached enzyme activity level in the blood after the procedure [33]. The reported 5-years survival rate after HSCT in 26 Japanese MPS II patients was 88.5% [33]. Kubaski et al. summarized the effects of HSCT in 27 newly described and reviewed 119 previously described patients [11]. Graft-versus-host disease occurred in 9% of published cases. None of the 27 newly described and nine of the previously published 119 patients died of transplant-associated complications, seven of those received unrelated bone marrow donor's transplant. This evidence calculates an 8% mortality rate of HSCT among published MPS II cases from 1984 to 2016 [11]. Improved outcomes are due to advanced conditioning regimens and better engraftment rates using pharmacokinetic analysis-guided, myeloablative busulfan depletion [37].

These data are essential to counsel the families when deciding for or against HSCT as caregivers take responsibility for the decision. The parents of the boy described as our Case 3 agreed on the HSCT, based on early age at presentation, low disease burden, and favorable cognitive abilities. Slovenia has a national pediatric transplantation center, but the experienced European transplantation centers are preferably chosen for the non-malignant indications. HSCT for metabolic diseases requires multidisciplinary and often international collaboration; therefore, UMC Utrecht, the Netherlands, was contacted [38]. HSCT with cord blood stem cells was successful at 22 months of age. Normal I2S enzyme activity in blood was measured after the engraftment. As Shapiro and Eisengart conclude from their review studies of cognitive outcomes after HSCT, quantitative neurocognitive results are missing [27]. The case reports found benefits of HSCT for somatic symptoms, while the beneficial effect on cognition was not proven. We could not directly compare the cognitive outcome of our patient at different age points (Fig. 1) as various cognitive measures were used. However, the results show that the patient's cognitive development is slowly progressing after treatment, although a significant cognitive and language impairment with attention and behavior problems remain.

HSCT is not an optimal treatment for MPSs, and the research is ongoing. Progressive improvements in techniques for genetic correction of autologous HSCs are also promising for MPS patients [39].

The main study limitation is its retrospective design; the therapy selection, tests and measurements were not basing on a pre-designed protocol, thus impeding the direct comparisons between the cases presented and also any strong conclusions. The missing information might come from multi-centre prospective studies which are however, very difficult to be performed in any ultra-rare diseases.

## 4 Conclusions

The three case presentations are opening the ongoing discussions about the treatment options in MPS II. ERT has some severe limitations: total financial costs, limited effectiveness of the therapy due to development of neutralizing antibodies, lack of blood-brain barrier passage, and limited penetration and effect in specific tissues. On the other hand, ERT is less invasive and has fewer risks comparing to HSCT. Current benefits of HSCT are also limited, but advanced conditioning regimens had improved the outcomes, and early HSCT is considered an effective treatment option for MPS II patients.

The challenges in the development of better treatment options in MPSs remain. Autologous HSCT using gene therapy may provide an improvement by reducing allogeneic treatment-related toxicities and by improving efficacy through augmented graft enzyme delivery [40]. Currently, MPS II has not been introduced in the neonatal screening programs in European countries; however, better treatment options will open additional discussions about neonatal screening for MPSs, as only pre-symptomatic management speculate successful outcomes for these patients [[40], [41], [42]].
